# PM_2.5_ Exposure-Linked Mitochondrial Dysfunction Negates SB216763-Mediated Cardio-Protection against Myocardial Ischemia–Reperfusion Injury

**DOI:** 10.3390/life13112234

**Published:** 2023-11-20

**Authors:** Bhavana Sivakumar, Ahmed Nadeem, Mashooq Ahmad Dar, Gino A. Kurian

**Affiliations:** 1 Vascular Biology Lab, School of Chemical and Biotechnology, SASTRA Deemed University, Thanjavur 613401, Tamil Nadu, India; bhavana@scbt.sastra.ac.in; 2 Department of Pharmacology and Toxicology, College of Pharmacy, King Saud University, Riyadh 11451, Saudi Arabia; 3 Laboratory of Preclinical Testing of Higher Standard, Nencki Institute of Experimental Biology of Polish Academy of Sciences 3, 02-093 Warsaw, Poland; m.dar@nencki.edu.pl

**Keywords:** PM_2.5_, GSK 3β, SB216763, cardiotoxicity, myocardial ischemia–reperfusion injury, mitochondria, oxidative stress

## Abstract

GSK3β is a promising target for treating various disease conditions, including myocardial ischemia–reperfusion injury (IR). This study investigated the potential of GSK3β as a novel drug for managing IR in rats exposed to PM_2.5_ for 1 day and up to 21 days. Female Wistar rats were exposed to PM_2.5_ at a concentration of 250 µg/m^3^ for 3 h daily for either a single day or 21 days. After exposure, the isolated rat hearts underwent 30 min of ischemia followed by 60 min of reperfusion. GSK3β inhibition effectively reduced IR injury in rat hearts from animals exposed to PM_2.5_ for 1 day but not in those exposed for 21 days. PM_2.5_ exposure disrupted the redox balance in mitochondria and reduced the gene expression of antioxidants (glutaredoxin and peroxiredoxin) and NRF2, which protects against oxidative stress. PM_2.5_ also impaired mitochondrial bioenergetics, membrane potential, and quality control, leading to mitochondrial stress. Importantly, PM_2.5_ increased the translocation of GSK3β into mitochondria and compromised the overall mitochondrial function, particularly in the 21-day-exposed rat myocardium. The results indicate that extended exposure to PM_2.5_ leads to oxidative stress that disrupts mitochondrial function and diminishes the effectiveness of GSK3β inhibitors in offering cardio-protection through mitochondria.

## 1. Introduction

Particulate matter (PM_2.5_) has become a major environmental concern worldwide due to its detrimental effects on human health [1]. PM_2.5_ is reported to augment the cardiovascular risk for arrhythmias, infarction, stroke, and even heart failure [2]. The molecular mechanism of the pathology is associated with oxidative stress, inflammation, and mitochondrial dysfunction. Pharmacological interventions targeting PM_2.5_-induced health effects have gained significant attention in recent times. The unique composition and toxicity of PM_2.5_ particles necessitate the development of specific pharmacological approaches to counteract their detrimental effects on human health. Various therapeutic targets are being explored, including antioxidants, anti-inflammatory agents, and drugs targeting specific molecular pathways implicated in PM_2.5_-induced toxicity [3,4,5,6,7,8]. Antioxidants, such as N-acetylcysteine (NAC) and vitamin C, have shown promise in reducing oxidative stress and mitigating the pro-inflammatory effects in patients [9]. Anti-inflammatory agents, such as corticosteroids and nonsteroidal anti-inflammatory drugs (NSAIDs), are being investigated for their potential to alleviate airway inflammation and respiratory symptoms [10]. However, none of these agents were explored to evaluate the potential to attenuate PM_2.5_-associated cardiotoxicity.

However, in recent years, targeting glycogen synthase kinase 3β (GSK3β) has shown significant promise as a therapeutic approach for cardiovascular diseases, particularly in the context of myocardial ischemia–reperfusion injury (IR) [11]. GSK3β has emerged as a crucial player in the pathogenesis of IR [12,13]. Interestingly, GSK3β exhibits distinct functions in response to different cellular conditions. For example, its activation during ischemia exacerbates myocardial injury, whereas its inhibition during reperfusion offers protective effects [13]. GSK3β inhibition has been shown to reduce infarct size, improve myocardial contractility, and attenuate apoptosis and inflammation in various preclinical models of IR [14,15]. One advantage of targeting GSK3β is its involvement in multiple signaling pathways implicated in cell survival and death [11]. GSK3β regulates various downstream effectors, including glycogen synthase, β-catenin, nuclear factor kappa B (NF-κB), and apoptotic proteins [16,17]. By modulating these signaling pathways, GSK3β inhibition can promote cell survival, limit inflammation, and suppress apoptosis in the myocardium following ischemia and reperfusion. Small molecule inhibitors of GSK3β, such as SB216763 and TDZD-8, have been shown to attenuate myocardial injury and improve cardiac function in various animal models [18,19]. Additionally, GSK3β inhibitors are reported to activate the Nrf2-ARE signaling axis and thereby regulate oxidative stress in neurological diseases [20]. These findings have laid the foundation for further investigations and potential translation into clinical practice. However, its modulation and suitability as a therapeutic target in cardiac tissue to attenuate IR injury in the presence of PM_2.5_ is unknown.

Recent studies have indicated a potential link between GSK3β activity and mitochondrial function. GSK3β has been reported to regulate mitochondrial morphology, bioenergetics, and oxidative stress responses [21]. Furthermore, pharmacological modulation of GSK3β activity has been shown to influence mitochondrial function and redox balance [22]. Mitochondria are both a major source and target of reactive oxygen species (ROS) production, and excessive ROS generation during IR can damage mitochondrial components, including lipids, proteins, and DNA [23]. This oxidative damage further exacerbates mitochondrial dysfunction and contributes to myocardial injury [24]. Pharmacological inhibition of GSK3β reduces ROS production, restores antioxidant enzyme activity, and alleviates oxidative stress, thus protecting against IR-induced mitochondrial damage.

Despite extensive research on the role of GSK3β in cardiovascular diseases that include IR, there is a notable knowledge gap regarding its therapeutic potential in the context of PM_2.5_-exposed hearts. The specific effects of GSK3β inhibition on oxidative stress reduction and mitochondrial preservation in PM_2.5_-exposed hearts remain largely unexplored, prompting the need for further investigation. Despite the well-established role of GSK3β in cardiac pathology, the potential therapeutic targeting of GSK3β in PM_2.5_-exposed hearts remains an unexplored avenue of research.

The pathophysiology of PM_2.5_-induced cardiovascular damage is mostly influenced by oxidative stress and mitochondrial dysfunction. Given the regulatory role of GSK3β in oxidative stress and mitochondrial function, it is crucial to investigate whether GSK3β inhibition can effectively reduce oxidative stress and preserve mitochondrial integrity in the context of PM_2.5_ exposure. Addressing these questions will provide valuable insights into the therapeutic potential of GSK3β inhibition in mitigating the adverse effects of PM_2.5_ on cardiac health.

This study aims to bridge the existing knowledge gap by examining the effects of GSK3β inhibition in PM_2.5_-exposed hearts. The study will employ an experimental model involving exposure to PM_2.5_ and subsequent GSK3β inhibition using a specific pharmacological inhibitor. The effects of GSK3β inhibition on oxidative stress markers and mitochondrial function will be assessed. The findings of this study are expected to provide insights into the interplay between PM_2.5_ exposure, mitochondrial redox stress, and the therapeutic advantage of targeting GSK3β. In addition, this study could aid in the creation of novel strategies that reduce the harmful health impacts of exposure to air pollution, especially for those who are exposed to PM_2.5_ for extended periods of time.

## 2. Methods

### 2.1. Reagents

From the National Institute of Standards and Technology in Gaithersburg, MD, USA, SRM-2975 was acquired. Unless otherwise noted, all of the chemicals and reagents used in this study were purchased from Sigma-Aldrich in St. Louis, MO, USA.

### 2.2. Animal Procurement and Maintenance

The animal experiments were carried out at SASTRA Deemed University, Thanjavur, India, in compliance with the rules set forth by the Committee for the Purpose of Control and Supervision of Experiments on Animals (CPCSEA). These studies were authorized by the Institutional Animal Ethical Committee (IAEC) using reference number 752/SASTRA/IAEC/RPP. Additionally, the ARRIVE criteria are followed in the conduct of the study. A controlled environment consisting of a 12 h light/dark cycle, regulated temperature and humidity (23 ± 2 °C, 60–70%), and housing for female Wistar rats weighing between 200 and 250 g. For the course of the trial, the rats had unlimited access to food and water.

### 2.3. Experimental Procedure

**Figure 1:**
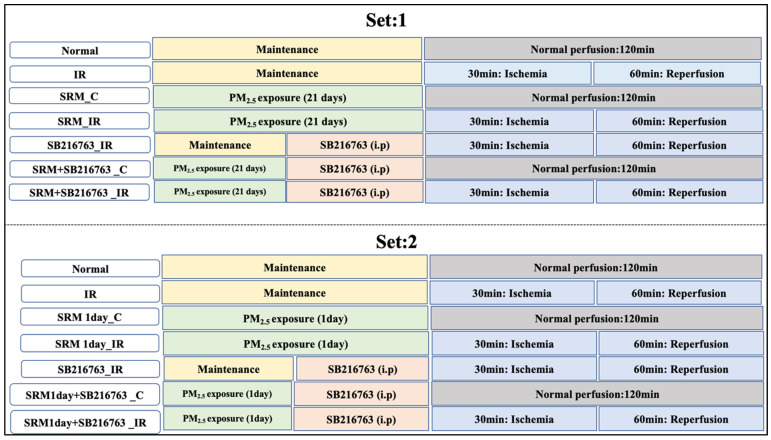
Study experimental groups.

A modified whole-body animal exposure model—detailed in our publication [25]—was used to expose the animals to PM_2.5_. To ensure accurate and reliable measurements, the pressure, temperature, and flow rate within the exposure chamber were carefully monitored and maintained at optimal levels throughout the experiment. By controlling these parameters, we aimed to create a controlled and consistent environment for the animals during the exposure period. We used a PRANA air-sourced CAIR air quality monitor to precisely determine the levels of PM, oxygen (O_2_), and carbon monoxide (CO), as well as the temperature and humidity inside the exposure chamber. We adopted two experimental approaches in the present study: (1) Animals were exposed to PM_2.5_ for 21 days. (2) Animals were exposed to PM_2.5_ for 1 day. Each experimental set was further divided into 7 groups as follows (Figure 1):

1: Animals exposed for 21 days: (1) Normal perfusion, (2) IR induction, (3) exposure to PM_2.5_ for 3 h every day for 21 days at a concentration of 250 µg/m^3^, (4) exposure to PM_2.5_ followed by IR induction, (5) intraperitoneal administration of SB216763, an inhibitor of GSK3β followed by IR induction, (6) for 21 days, 3 h a day of exposure to 250 µg/m^3^ PM_2.5_ was followed by the administration of 0.7 mg/kg of GSK3β inhibitor (SB216763) and 120 min of normal perfusion, and (7) for 21 days, 3 h a day of exposure to 250 µg/m^3^ PM_2.5_ was followed by the administration of 0.7 mg/kg of GSK3β inhibitor (SB216763) and IR induction.

2: Animals exposed for 1 day: (1) Normal perfusion, (2) IR induction, (3) exposure to PM_2.5_ at a concentration of 250 µg/m^3^ for 1 h, (4) PM_2.5_ exposure followed by IR induction, (5) intraperitoneal administration of SB216763, an inhibitor of GSK3β followed by IR induction, (6) after 1 h of exposure to 250 µg/m^3^ of PM_2.5_, 0.7 mg/kg of GSK3β inhibitor (SB216763) was administered, and normal perfusion was maintained for 120 min, and (7) after 1 h of exposure to 250 µg/m^3^ of PM_2.5_, 0.7 mg/kg of GSK3β inhibitor (SB216763) was administered followed by IR induction.

### 2.4. IR Induction and Hemodynamics Assessment

The ex vivo approach began with administering rats anesthesia using a combination of xylazine (20 mg/kg) and ketamine (80 mg/kg). After that, the rat’s heart was removed, and Krebs–Henseleit buffer—which had the following composition: 118.0 mM NaCl, 4.7 mM KCl, 1.9 mM CaCl_2_, 1.2 mM MgSO_4_, 25.0 mM NaHCO_3_, 1.2 mM KH_2_PO_4_, and 10.1 mM glucose—was used to start the perfusion process. The buffer had a pH of 7.4. This perfusion was carried out at a temperature of 37 °C and maintained with continuous oxygenation, combining 95% O_2_ and 5% CO_2_. A perfusion system (ADInstruments, Dunedin, New Zealand) was used for 20 min to support the heart, with a consistent perfusate pressure of 70 mmHg being maintained. Using a pressure transducer attached to a latex balloon positioned in the left ventricle of the heart, hemodynamic changes were closely observed. A PowerLab data acquisition device from ADInstruments was used to constantly capture electrical data at the same time. LabChart Pro 8 software was then used to analyze the data [25]. Using H&E staining, structural alterations in the myocardium were detected.

### 2.5. Western Blot

Using an ice-cold RIPA lysis solution, the samples were homogenized, and Lowry’s method was used to find the protein concentration. Following that, in SDS lysis buffer, equivalent protein quantities were made and denatured for 15 min at 80 °C. Afterward, the denatured proteins were separated using SDS-polyacrylamide gel electrophoresis (SDS-PAGE) with a 5% stacking gel and a 10% resolving gel staining, along with which the Western blot protein standard (Thermofisher # LC5602) was used for quality control. Onto PVDF membranes with a 0.45 μm pore size, the resolved proteins were transferred. Following the transfer of the bands onto membranes, Ponceau staining was employed to visualize the proteins residing on the membranes. Subsequently, we carefully selected only the required bands based on the molecular marker as our targets for further analysis. This approach was adopted with the explicit purpose of conserving antibodies and optimizing resource utilization, thereby minimizing the potential for background noise. To prevent non-specific binding, the membranes were blocked using 5% BSA in TBST (Tris-buffered saline with Tween) for 1 h. The membranes were thoroughly rinsed with TBST for a duration of 15 min, and then they were treated with primary antibodies obtained from Cell Signaling Technology for an overnight period at 4 °C. The primary antibodies included beta-actin (CST#13E5), parkin (CST#2132), MFF (CST#84580), COX IV (CST#4844), and GSK3B (CST #9315). We bought MFN1 (ab126575) from Abcam. A 1:2000 ratio was used to dilute the antibodies. After the primary antibody incubation, the membranes were treated with secondary antibodies (CST #7074) diluted at a ratio of 1:3000 in TBST for 1 h at room temperature. This was followed by three 15 min TBST washes. The blot membranes were photographed using a chemiluminescent detection system (ECL) in a Chemi-Doc XRS apparatus from BioRad, Hercules, CA, USA. For capturing high-quality images of the Western blots, we utilized Bio-Rad’s Quantity One (version 4.6) software. This software played a pivotal role in ensuring precise image acquisition and analysis. Specifically, it was used to crop the specific regions of the membrane housing the bands of interest and also to remove the background noise if any. This cropping process was executed with utmost precision to enhance visibility and clarity while preserving the original, unmodified blots, which are provided in the Supplementary Materials of the manuscript [26].

### 2.6. RNA Isolation and Real-Time PCR

TRIzol reagent (15596026, Thermo Scientific, Waltham, MA, USA) was used to isolate fresh heart tissues weighing 50–100 mg in accordance with the previously published protocol [27]. Using a Thermo Fisher nanodrop 2000 device, the extracted RNA’s concentration, purity, and integrity were evaluated. Thermo Scientific Verso cDNA Synthesis Kit (AB-1453/A) was used to carry out reverse transcription (RT). To initiate the RT reaction, a reaction mixture consisting of 4 µL of 5X cDNA synthesis buffer, 2 µL of dNTP mix, 1 µL of RNA primer (Oligo dT), 1 µL of RT Enhancer, and 1 µL of Verso Enzyme Mix was combined, resulting in a volume of 20 µL. A 30 min reaction at 42 °C and a 2 min cycle at 95 °C were required for the cDNA synthesis procedure. The Dynamo Flash SYBR green kit (#F415S, Thermo Scientific, USA) was then used for quantitative polymerase chain reaction (qPCR) on an ABI 7500 thermocycler (Applied Biosystems, Waltham, MA, USA) for the Real-Time PCR System. The housekeeping gene for standardization was beta-actin, and the mRNA expression levels were standardized using the 2^−ΔΔCt^ technique. A 1 min initial denaturation step at 95 °C was followed by 40 cycles of denaturation at 95 °C for 15 s, annealing for 20 s at the optimal temperature, and elongation for 20 s at 72 °C in the qPCR amplification procedure. The last stage of the extension was performed at 72 °C for 1 min to wrap up the program. Table 1 provides a list of primers used in the course of the study.

### 2.7. Mitochondrial Isolation and Respiration Assessment

In summary, an isolation buffer containing 220 mM mannitol, 70 mM sucrose, 5 mM MOPS, 2 mM EDTA, and 0.2% BSA was used for tissue lysis in order to generate a 10% cardiac homogenate. The nuclear fraction was then separated by centrifuging the homogenate at 500× *g* for 10 min at 4 °C. Following another round of centrifugation at 12,000× *g* for 10 min at 4 °C, the resultant supernatant was subjected to lysosomes and light and heavy mitochondria, yielding a crude mitochondrial fraction. A sucrose density gradient was used to further separate the unprocessed mitochondrial pellet. We created a gradient in a 5 mL centrifuge tube by layering sucrose densities of 1.25, 1.22, 1.19, 1.15, 1.11, and 1.09 g/cm^3^ in PBS at a pH of 7.4. Re-suspended in PBS, the pellet containing crude mitochondria was then positioned on top of the density gradient and centrifuged using a swingout bucket rotor set to the lowest acceleration and deceleration settings for 3 h at 4 °C (100,000× *g*). This separation resulted in the division of lysosomes into two separate fractions: 1.12 g/cm^3^ of lysosomes and 1.18 g/cm^3^ of mitochondria. After harvesting the mitochondria fraction, we stored it in a pH 7.4-adjusted storage solution with 0.25 M sucrose, 1 mM EDTA, and 10 mM HEPES [28].

A Clarke-type oxygen electrode (Hansatek, England, UK) was used to monitor the oxygen consumption rate at 37 °C in order to evaluate the mitochondrial respiration capacity. This measurement was performed in a respiration buffer that contained different substrates to stimulate respiration mediated by complex I and complex II. The substrates used included glutamate/malate (GM) at concentrations of 5 mM and 2.5 mM, as well as succinate at a concentration of 5 mM. The integrity of the isolated mitochondria was evaluated by calculating the respiratory control ratio (RCR), which represents the ratio of state 3 to state 4 respiration. Furthermore, the state 3 and state 4 respiration rates were assessed following the addition of 2.5 mM ADP and 2,4-dinitrophenol. These measurements allowed for the assessment of the efficiency of oxidative phosphorylation, a critical process in mitochondrial function. The ADP/O ratio, which represents the ratio of ADP utilized to the amount of oxygen consumed during oxidative phosphorylation, was used as a measure of the efficiency of this process.

### 2.8. Oxidative Stress Assessment

The activity of superoxide dismutase (SOD) and catalase, as well as the levels of GSH/GSSG (glutathione reduced/oxidized) in mitochondria, was determined using the established methods described in a previous publication [29]. Additionally, the gene expression of mitochondrial antioxidants such as glutaredoxin 1, peroxiredoxin 3, peroxiredoxin 6, and NRF2 was analyzed via RT PCR. To assess the levels of reactive oxygen species (ROS) generation, the indicator dichlorodihydrofluorescein diacetate (DCHFDA) was utilized. Spectrophotometric analysis was employed to measure the fluorescence of DCHFDA in the mitochondria. The mitochondrial samples were treated with 5 μM of DCHFDA, and the fluorescence was recorded at 503 nm for excitation and 529 nm for emission.

The level of lipid peroxidation was assessed using a thiobarbituric acid reactive species (TBARS) assay that is based on the generation of malondialdehyde. The sample was added to a reaction mixture containing 15% trichloroacetic acid and 0.8% thiobarbituric acid, adjusted to pH 3.5, and incubated for 1 h at 75 °C in a water bath. The pink complex was extracted using a 15:1 butanol-to-pyridine mixture after cooling, and the absorbance of the organic layer was measured at 532 nm. In the experiment, malondialdehyde served as the standard.

After homogenizing the tissues, the samples were added to 4.0 mL of 10 mM 2,4-dinitrophenylhydrazine (DNPH) in 2.5 M HCl for the protein carbonyl test. The solutions were then incubated for an hour at room temperature in the dark, shaking them occasionally every 15 min. Each tube was filled with 5 mL of 20% TCA (*w*/*v*), and the mixture was then allowed to sit on ice for 10 min. The supernatant was disposed of after centrifuging the tubes for 20 min at 3500 rpm in order to remove the protein pellet. Following that, a second wash using 10% TCA was carried out as was previously indicated. Ultimately, the precipitates were rinsed three times with 4 mL of ethanol:ethyl acetate (1:1, *v*/*v*) in order to eliminate any last traces of lipids and DNPH. The resulting protein pellet was dissolved in 2 mL of 6 M guanidine hydrochloride after being incubated at 37 °C for 10 min. The insoluble components were then separated using centrifugation. To measure the carbonyl concentration, the spectra of representative samples were taken between 355 and 390 nm.

### 2.9. Inductively Coupled Mass Spectrophotometry Analysis

Metal bioaccumulation levels in mitochondria resulting from PM exposure were assessed using inductively coupled plasma mass spectrometry (ICP-MS). The samples were initially dried at 95 °C for 24 h, followed by digestion with HNO_3_ at 90 °C for 4 h. Subsequently, the digested samples were diluted with MilliQ water to achieve a final HNO3 concentration of 3%. The quantification of metals present in the digested samples was performed using ICP-MS (ELAN 6000, PerkinElmer-Sciex, Waltham, MA, USA), which enables accurate measurement of metal concentrations. The resulting values were expressed in parts per million (PPM), providing information on the amount of metal bioaccumulation in the mitochondria.

### 2.10. Statistical Evaluation

The data are presented as the mean ± standard deviation (SD). To evaluate the differences between groups, a statistical analysis was performed using one-way analysis of variance (ANOVA) followed by Dunnett’s test as a post hoc analysis, where *^$^ *p* < 0.05 is considered significant. The statistical analysis was conducted using GraphPad Prism 7.0 software.

## 3. Results

### 3.1. GSK3β Inhibitor Failed to Improve Cardiac Function in Rat Hearts Exposed to PM_2.5_ for 21 Days and Subjected to IR

**Figure 2:**
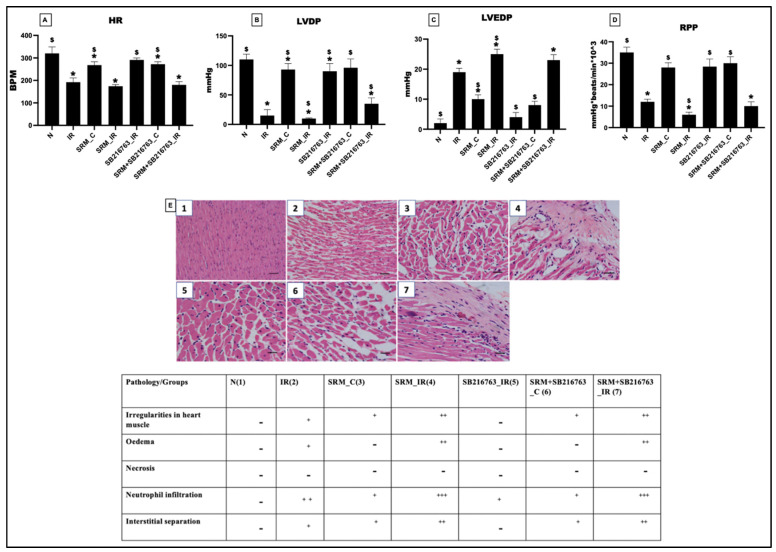
Evaluation of cardiac hemodynamic parameters using Lab Chart software. (**A**) Heart rate (HR), (**B**) left ventricular developed pressure (LVDP), (**C**) left ventricular end-diastolic pressure (LVEDP), and (**D**) rate pressure product (RPP). (**E**) H&E staining of the myocardium and injury score (* *p* < 0.05 vs. N, ^$^ *p* < 0.05 vs. IR). Groups: (1) Animals underwent normal perfusion (normal), (2) animals underwent the standard IR procedure of 30 min of ischemia and 60 min of reperfusion (IR control), (3) animals were exposed to PM_2.5_ for 21 days after which the hearts were isolated and subjected to normal perfusion, (4) animals were exposed to PM_2.5_ for 21 days after which the hearts were isolated and subjected to IR procedure, (5) animals were administered SB216763 intraperitoneally after which the hearts were isolated and subjected to IR procedure, (6) animals were exposed to PM_2.5_ for 21 days and administered SB216763 intraperitoneally after which the hearts were isolated and subjected to normal perfusion, (7) animals were exposed to PM_2.5_ for 21 days and administered SB216763 intraperitoneally after which the hearts were isolated and subjected to IR procedure (6 animals in each group). + is calculated based on the severity of cardiac injury.

The heart rate (HR), left ventricular developed pressure (LVDP), left ventricular end-diastolic pressure (LVEDP), and rate pressure product (RPP) from the normal (N) and IR control were all reduced after 21 days of exposure to PM_2.5_. In the SRM_C group, there were significant reductions of 16% in HR, 15% in LVDP, and 20% in RPP, while LVEDP increased by a substantial 80%. When SB216763 was introduced in the SRM + SB216763_C group, there were only marginal improvements in cardiac hemodynamics, with HR increasing by 2%, LVDP by 3%, RPP by 6%, and LVEDP by 2%. HR, RPP, and LVDP decreased by 10%, 33%, and 50% in comparison to the IR control group after the induction of IR, worsening the hemodynamic parameter decrease in the SRM_IR group. However, it is important to note that the administration of SB216763 did not yield any notable improvements in the hemodynamics of PM_2.5_-exposed hearts subjected to IR (Figure 2).

### 3.2. GSK3β Inhibitor Failed to Improve Mitochondrial Respiration and ATP Levels in Rat Hearts Exposed to PM_2.5_ for 21 Days and Subjected to IR

**Figure 3:**
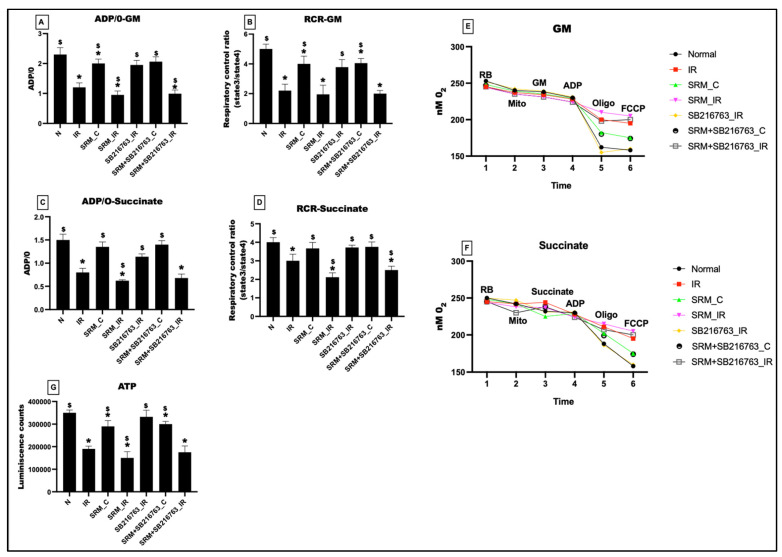
The oxygraph-measured respiratory status of the mitochondria. (**A**) ADP/O ratio in the glutamate–malate (GM) medium, (**B**) respiratory control ratio (RCR) in the GM medium, (**C**) ADP/O ratio in the succinate medium, and (**D**) RCR in the succinate medium. Representative traces of mitochondrial respiration in different experimental groups were measured via oxygraphy using (**E**) GM as substrate, (**F**) succinate as substrate, and (**G**) ATP levels in the mitochondrial samples isolated from heart tissues (* *p* < 0.05 vs. N, ^$^ *p* < 0.05 vs. IR). Groups: (1) Animals underwent normal perfusion (normal), (2) animals underwent the standard IR procedure of 30 min of ischemia and 60 min of reperfusion (IR control), (3) animals were exposed to PM_2.5_ for 21 days after which the hearts were isolated and subjected to normal perfusion, (4) animals were exposed to PM_2.5_ for 21 days after which the hearts were isolated and subjected to IR procedure, (5) animals were administered SB216763 intraperitoneally after which the hearts were isolated and subjected to IR procedure, (6) animals were exposed to PM_2.5_ for 21 days and administered SB216763 intraperitoneally after which the hearts were isolated and subjected to normal perfusion, and (7) animals were exposed to PM_2.5_ for 21 days and administered SB216763 intraperitoneally after which the hearts were isolated and subjected to IR procedure (6 animals in each group).

Figure 3 illustrates how exposure to PM_2.5_ for 21 days decreased the mitochondrial respiration efficiency of the myocardium. The ADP/O ratio in the presence of glutamate–malate (GM) and succinate-energized respiratory media showed a 13% and 10% decline in PM_2.5_-exposed heart, which was further declined to 59% and 58%, respectively, in the presence of IR. The corresponding RCR values for the GM and succinate respiration buffer showed a decline of 20% and 8% in PM_2.5_-exposed hearts and a respective decline of 61% and 47% in hearts subjected to PM_2.5_-induced IR. However, administration of the GSK3β inhibitor resulted in no significant improvement in ADP/O levels or the RCR ratios in rats exposed to PM_2.5_ for 21 days, or in rats exposed to PM_2.5_ for 21 days followed by IR, compared to their respective controls.

The ATP levels showed a significant decline of 18% in PM_2.5_-exposed myocardium (SRM_C). In the presence of IR, the ATP levels declined to 57% (vs. normal). GSK3β inhibitor could improve the ATP levels only by 3% (group 6: rats exposed to PM_2.5_ for 21 days) and 14% (group 7: rats exposed to PM_2.5_ for 21 days followed by IR), respectively.

### 3.3. GSK3β Inhibitor Unable to Improve the Expression Levels of Mitochondrial Quality Control Genes in Rat Hearts Exposed to PM_2.5_ for 21 Days and Subjected to IR

**Figure 4:**
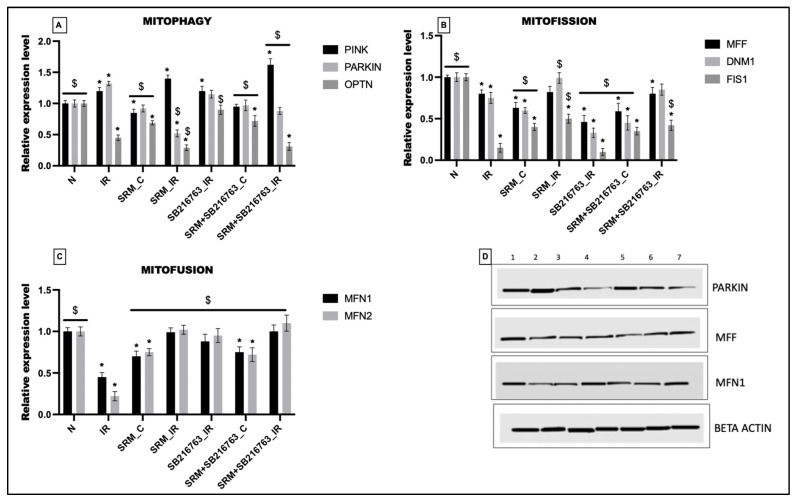
mRNA expression levels of (**A**) mitophagy genes (PINK, PARKIN, and OPTN), (**B**) mitofission genes (MFF, DNM1, and FIS1), and (**C**) mitofusion genes (MFN1, MFN2) (* *p* < 0.05 vs. Normal). (**D**) Protein expression levels of Parkin, MFF, and MFN1 were assessed via Western blot (1: animals underwent normal perfusion (normal), 2: animals underwent the standard IR procedure of 30 min of ischemia and 60 min of reperfusion (IR control), 3: animals were exposed to PM_2.5_ for 21 days after which the hearts were isolated and subjected to normal perfusion, 4: animals were exposed to PM_2.5_ for 21 days after which the hearts were isolated and subjected to IR procedure, 5: animals were administered SB216763 intraperitoneally after which the hearts were isolated and subjected to IR procedure, 6: animals were exposed to PM_2.5_ for 21 days and administered SB216763 intraperitoneally after which the hearts were isolated and subjected to normal perfusion, 7: animals were exposed to PM_2.5_ for 21 days and administered SB216763 intraperitoneally after which the hearts were isolated and subjected to IR procedure) (* *p* < 0.05 vs. N, ^$^ *p* < 0.05 vs. IR).

The effect of PM_2.5_ exposure on the expression of mitochondrial quality genes—genes related to mitophagy, fission, and fusion—is shown in Figure 4. Twenty-one days of PM_2.5_ exposure induced a 15%, 8%, and 31% decline in the expression of mitophagy genes like PINK, PARKIN, and OPTN, respectively, compared to the normal control. However, administration of GSK3β inhibitor improved the expression of these genes insignificantly by 10%, 5%, and 4% (vs. group 3: rats exposed to PM_2.5_ for 21 days). Similarly, the mitochondrial fission genes like MFF, DNM1, and FIS1 showed 37%, 41%, and 60% decline, respectively, in the PM_2.5_ exposure group. A similar trend was observed in the expression of mitochondrial fusion genes (MFN1 and MFN2) as well. As observed in SRM + SB216763_C, GSK3β inhibitor insignificantly reduced the expression of MFF, DNM1, and FIS1 by 6%, 23%, and 13%, respectively (vs. group SRM_C). Similarly in group SRM + SB216763_C, the expression of MFN1 and MFN2 showed an insignificant upregulation compared to group SRM_C.

IR induction upregulated the expression of PINK by 29% and reduced the expression of PARKIN and OPTN by 48% and 71%, compared to the normal control in the SRM_IR group (PM_2.5_-exposed rat heart subjected to IR). However, in comparison with the IR control group, we observed an insignificant upregulation of PINK, followed by a significant downregulation of PARKIN and OPTN. Administration of GSK3β inhibitor increased the expression of PINK, PARKIN, and OPTN by 14%, 40%, and 6% in group SRM + SB216763_IR (vs. SRM_IR). Analysis of mitochondrial fission and fusion genes showed an 18%, 1%, and 50% decline in the expression of MFF, DNM1, and FIS1, respectively, in SRM_IR (vs. normal). Even the expression of MFN1 showed an insignificant decline of 1% and MFN2 showed an upregulation of 1% (vs. normal) in the SRM_IR group. However as observed in SRM + SB216763_IR, GSK3β inhibitor insignificantly reduced the expression of MFF, DNM1, and FIS 1 by 2%, 14%, and 16% (vs. SRM_IR) and insignificantly increased the expression of MFN1 and MFN2 by 1% and 7%, respectively.

### 3.4. GSK3β-Inhibitor-Mediated Improvement Was Absent in the Expression of Mitochondrial PGC 1α, TFAM, and POLG Genes in Rat Hearts Exposed to PM_2.5_ for 21 Days and Subjected to IR

**Figure 5:**
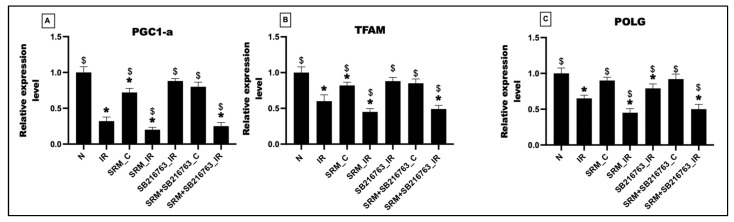
mRNA expression levels of (**A**) PGC1-a, (**B**) TFAM, and (**C**) POLG. Groups: (1) Animals underwent normal perfusion (normal), (2) animals underwent the standard IR procedure of 30 min of ischemia and 60 min of reperfusion (IR control), (3) animals were exposed to PM_2.5_ for 21 days after which the hearts were isolated and subjected to normal perfusion, (4) animals were exposed to PM_2.5_ for 21 days after which the hearts were isolated and subjected to IR procedure, (5) animals were administered SB216763 intraperitoneally after which the hearts were isolated and subjected to IR procedure, (6) animals were exposed to PM_2.5_ for 21 days and administered SB216763 intraperitoneally after which the hearts were isolated and subjected to normal perfusion, and (7) animals were exposed to PM_2.5_ for 21 days and administered SB216763 intraperitoneally after which the hearts were isolated and subjected to IR procedure (6 animals in each group) (* *p* < 0.05 vs. N, ^$^ *p* < 0.05 vs. IR).

As observed in Figure 5, exposure to PM_2.5_ for 21 days resulted in a 28% decline in the expression of the master regulator gene like PGC 1α, whereas TFAM and POLG gene expression was decreased by 18% and 10%, respectively, in group 3 compared to normal control. Administration of GSK3β inhibitor insignificantly improved the basal level expression of these genes by 10%, 3%, and 2% in group 6, respectively (vs. SRM_C). IR induction further declined the expression level of PGC1α, TFAM, and POLG by 80%, 55%, and 55% (as observed in SRM_IR), which was insignificantly improved to 20%, 8%, and 10% by GSK3β inhibitor in group SRM + SB216763_IR compared to group SRM_IR.

### 3.5. Elevated Oxidative Stress in Rat Hearts Exposed to PM_2.5_ for 21 Days and Subjected to IR Persists Even with the Administration of GSK3β Inhibitor

PM_2.5_ exposure for 21 days resulted in a 15% and 37% decrease in the levels of antioxidant enzymes like SOD and catalase and a 22% decrease in GSH/GSSG levels in SRM_C. The ROS level measured via DCFHDA showed a 15% increase in the SRM_C group. In support of these findings, the gene expression of antioxidant proteins like glutaredoxins, peroxiredoxins, and NRF2 was significantly reduced compared to the normal control. However, administration of GSK3β inhibitor resulted in an insignificant increase in the levels of SOD and catalase by 6% and 12% and the GSH/GSSG ratio by 7%, respectively (vs. SRM_C). The ROS levels were insignificantly reduced by 11% and the expression of antioxidant genes showed an insignificant increase at the basal level.

**Figure 6:**
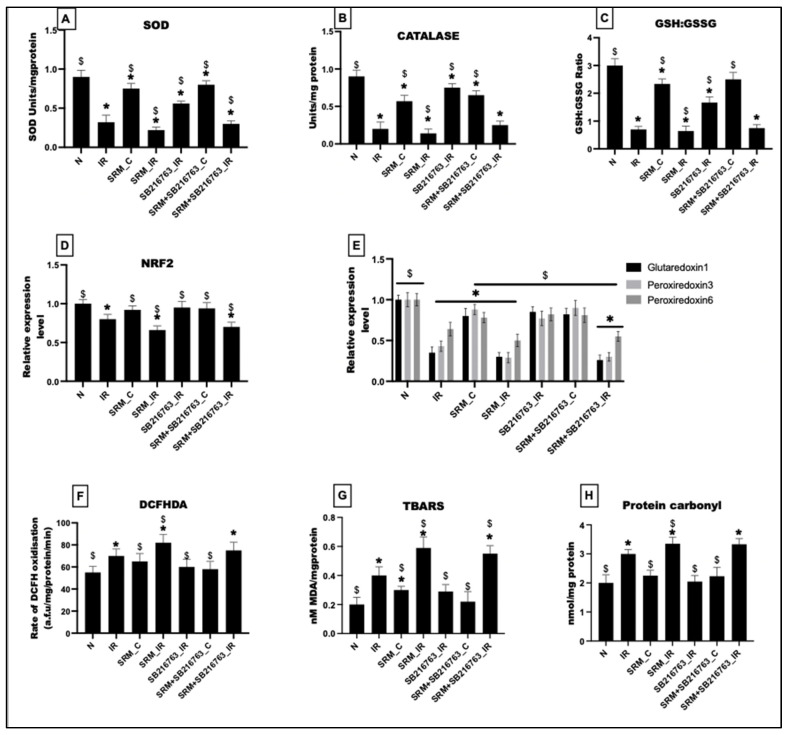
Assessment of mitochondrial oxidative stress: (**A**) SOD, (**B**) catalase, (**C**) GSH:GSSG ratio, and (**D**) DCFHDA assay for ROS detection. mRNA expression of (**E**) NRF2, (**F**) glutaredoxin 1, peroxiredoxin 3,6, (**G**) lipid peroxidation assessment via TBARS assay, and (**H**) protein carbonyl assay (* *p* < 0.05 vs. N, ^$^ *p* < 0.05 vs. IR). Groups: (1) Animals underwent normal perfusion (normal), (2) animals underwent the standard IR procedure of 30 min of ischemia and 60 min of reperfusion (IR control), (3) animals were exposed to PM_2.5_ for 21 days after which the hearts were isolated and subjected to normal perfusion, (4) animals were exposed to PM_2.5_ for 21 days after which the hearts were isolated and subjected to IR procedure, (5) animals were administered SB216763 intraperitoneally after which the hearts were isolated and subjected to IR procedure, (6) animals were exposed to PM_2.5_ for 21 days and administered SB216763 intraperitoneally after which the hearts were isolated and subjected to normal perfusion, and (7) animals were exposed to PM_2.5_ for 21 days and administered SB216763 intraperitoneally after which the hearts were isolated and subjected to IR procedure (6 animals in each group).

As observed in group SRM_IR, IR induction significantly reduced the SOD, catalase, and GSH:GSSG levels in the PM_2.5_-exposed group by 76%, 84%, and 78%, respectively, compared to normal control. The ROS levels showed a significant increase of 33% and the gene expression of NRF2 along with glutaredoxins and peroxiredoxins was significantly downregulated. GSK3β inhibitor increased the antioxidant levels (SOD, catalase, GSH:GSSG) by 26%, 44%, and 15%, respectively (vs. SRM_IR). The ROS levels were also insignificantly reduced (8%) in SRM + SB216763_IR compared to SRM_IR. In support of the above findings, GSK3β inhibitor administration could not recover the expression of antioxidant genes like NRF2, glutaredoxin, and peroxiredoxin in PM_2.5_-exposed IR-induced rat hearts (Figure 6). In support of the above findings, we found a significant increase in the lipid peroxidation levels in the IR control group, which was significantly increased in the presence of PM_2.5_ exposure (SRM_IR). GSK3β inhibitor treatment, however, was unable to lower the levels of lipid peroxidation in SRM_IR hearts. Likewise, in SRM_IR groups, there was a notable increase in protein carbonyl levels that did not return to baseline levels when the inhibitor was present.

### 3.6. GSK3β Inhibitor Failed to Improve the Expression of Mitochondrial Detoxification Genes

**Figure 7:**
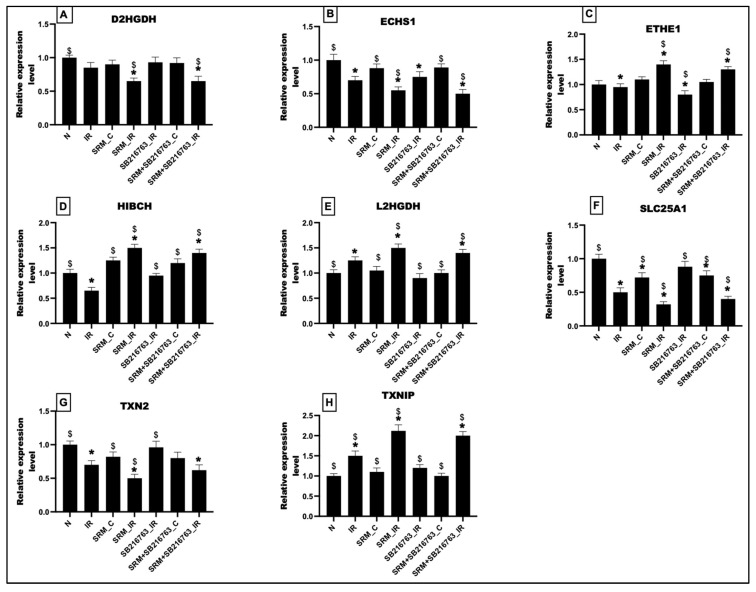
mRNA levels of (**A**) D2HGDH, (**B**) ECHS1, (**C**) ETHE1, (**D**) HIBCH, (**E**) L2HGDH, (**F**) SLC25A1, (**G**) TXN2, and (**H**) TXNIP (* *p* < 0.05 vs. N, ^$^ *p* < 0.05 vs. IR). Groups: (1) Animals underwent normal perfusion (normal), (2) animals underwent the standard IR procedure of 30 min of ischemia and 60 min of reperfusion (IR control), (3) animals were exposed to PM_2.5_ for 21 days after which the hearts were isolated and subjected to normal perfusion, (4) animals were exposed to PM_2.5_ for 21 days after which the hearts were isolated and subjected to IR procedure, (5) animals were administered SB216763 intraperitoneally after which the hearts were isolated and subjected to IR procedure, (6) animals were exposed to PM_2.5_ for 21 days and administered SB216763 intraperitoneally after which the hearts were isolated and subjected to normal perfusion, and (7) animals were exposed to PM_2.5_ for 21 days and administered SB216763 intraperitoneally after which the hearts were isolated and subjected to IR procedure (6 animals in each group).

As observed in Figure 7, PM_2.5_ exposure for 21 days deteriorated the expression pattern of mitochondrial genes that react to toxic substances. At the basal level, genes like D2HGDH, ECHS1, SLC25A1, and TXN2 were downregulated. However, genes like ETHE1, HIBCH, L2HGDH, and TXNIP were upregulated compared to normal control. In fact, most of these changes were insignificant except in SLC25A1. There were no notable alterations in the expression patterns of these genes in the SRM_C group, following the administration of a GSK3β inhibitor. The expression of ETHE1, HIBCH, L2HGDH, and TXNIP significantly increased with IR induction, whereas the expression of D2HGDH, ECHS1, SLC25A1, and TXN2 significantly decreased compared to normal. Administration of GSK3β inhibitor failed to significantly restore the expression pattern of these genes to normal levels. Interestingly, SRM_IR and SRM + SB216763_IR showed similar changes in the gene expression of all enzymes, indicating that the presence of GSK3β inhibitor did not influence the PM_2.5_-associated modulation of these genes, which adversely affects the detoxifying capacity of mitochondria.

### 3.7. Protein Expression Level of p-GSK3β and GSK3β in Cardiac Tissue and Mitochondria from Rats Exposed to PM_2.5_ for 21 Days in the Presence and Absence of GSK3β Inhibitor

**Figure 8:**
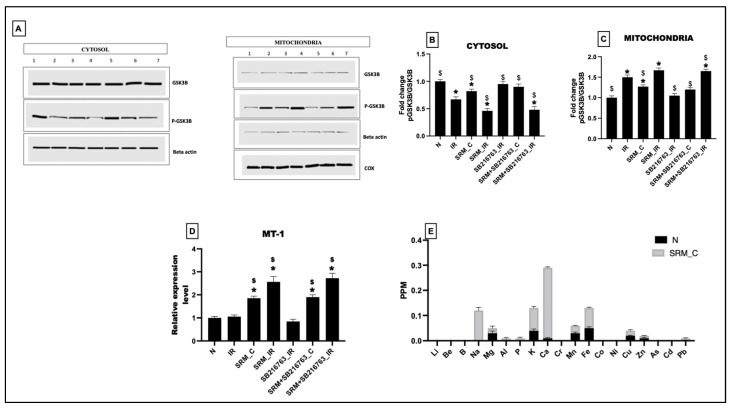
(**A**) Protein expression of GSK3β and p-GSK3β (1: normal perfusion, 2: IR control, 3: PM_2.5_ exposure for 21 days followed by normal perfusion, 4: PM_2.5_ exposure for 21 days followed by IR induction, 5: GSK3β inhibitor administration followed by IR induction, 6: PM_2.5_ exposure followed by GSK3β inhibitor administration and normal perfusion, and 7: PM_2.5_ exposure followed by GSK3β inhibitor administration and IR induction). Quantification of proteins in (**B**) cytosol, (**C**) mitochondria, (**D**) mRNA level expression of MT-1 (* *p* < 0.05 vs. N, ^$^ *p* < 0.05 vs. IR), and (**E**) ICPMS data showing metal deposition in isolated mitochondria. Groups: (1) Animals underwent normal perfusion (normal), (2) animals underwent the standard IR procedure of 30 min of ischemia and 60 min of reperfusion (IR control), (3) animals were exposed to PM_2.5_ for 21 days after which the hearts were isolated and subjected to normal perfusion, (4) animals were exposed to PM_2.5_ for 21 days after which the hearts were isolated and subjected to IR procedure, (5) animals were administered SB216763 intraperitoneally after which the hearts were isolated and subjected to IR procedure, (6) animals were exposed to PM_2.5_ for 21 days and administered SB216763 intraperitoneally after which the hearts were isolated and subjected to normal perfusion, and (7) animals were exposed to PM_2.5_ for 21 days and administered SB216763 intraperitoneally after which the hearts were isolated and subjected to IR procedure (6 animals in each group).

As observed in Figure 8A–C, total GSK3β proteins were relatively similar across the experimental groups in both cytosolic and mitochondrial fractions. Cytosolic fraction showed a significant decline in the protein level of p-GSK3β in groups 2: IR, 3: SRM_C, 4: SRM_IR, and 7: SRM + SB216763_IR. A corresponding increased protein level of GSK3β in mitochondrial fractions of groups 2, 3, 4, and 7 indicates that under mitochondrial stress, it facilitates the translocation of p-GSK3β into mitochondria and regulates its function.

Further analysis at the mRNA level demonstrated a significant increase in MT-1 expression at the basal level in group SRM_C. IR induction significantly increased the expression of MT-1 compared to the normal control, which could not be restored to normal levels with the administration of a GSK3β inhibitor. Figure 7D displays the ICPMS data of metal deposition in the mitochondria of normal and PM_2.5_-exposed (SRM_C) groups. Metals like Na, Mg, Ca, Al, P, K, Mn, Fe, Cu, Zn, and Pb showed increased deposition in the mitochondria due to 21 days of exposure (Figure 8E).

### 3.8. GSK3β Inhibitor Improved Mitochondrial Respiration and ATP Levels and Attenuated Cardiac Injury and Facilitated Physiological Recovery in Single-Day PM_2.5_-Exposed Rat Hearts Subjected to IR

**Figure 9:**
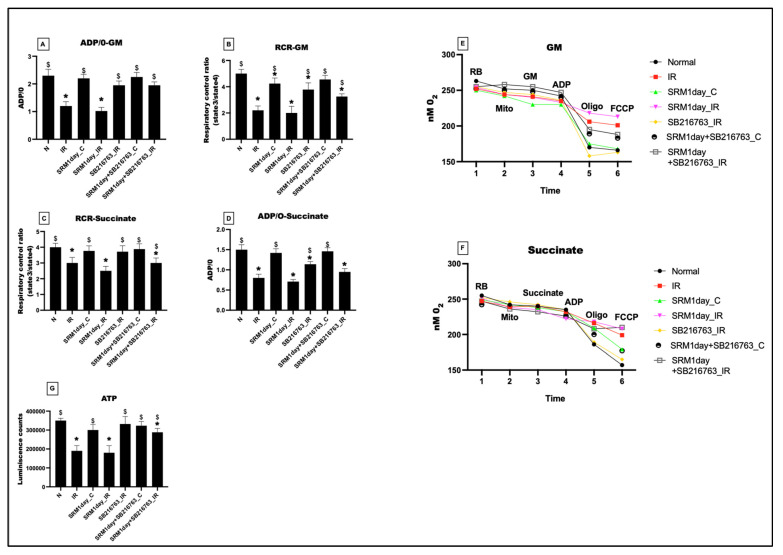
The mitochondrial respiratory status measured via oxygraph. (**A**) ADP/O ratio in the glutamate–malate (GM) medium, (**B**) respiratory control ratio (RCR) in the GM medium, (**C**) ADP/O ratio in the succinate medium, and (**D**) RCR in the succinate medium. Representative traces of mitochondrial respiration in different experimental groups were measured via oxygraphy using (**E**) GM as substrate, (**F**) succinate as substrate, and (**G**) ATP levels in the mitochondrial samples isolated from heart tissues (* *p* < 0.05 vs. N, ^$^ *p* < 0.05 vs. IR). Groups 1: Animals underwent normal perfusion (normal), 2: animals underwent the standard IR procedure of 30 min of ischemia and 60 min of reperfusion (IR control), 3: animals were exposed to PM_2.5_ for 21 days after which the hearts were isolated and subjected to normal perfusion, 4: animals were exposed to PM_2.5_ for 1 day after which the hearts were isolated and subjected to IR procedure, 5: animals were administered SB216763 intraperitoneally after which the hearts were isolated and subjected to IR procedure, 6: animals were exposed to PM_2.5_ for 1 day and administered SB216763 intraperitoneally after which the hearts were isolated and subjected to normal perfusion, and 7: animals were exposed to PM_2.5_ for 1 day and administered SB216763 intraperitoneally after which the hearts were isolated and subjected to IR procedure (6 animals in each group).

As observed in Figure 9, a single exposure to PM_2.5_ resulted in no significant changes in ADP/O in both GM and succinate-energized medium in SRM1day_C compared to normal control. However, IR induction reduced the respiratory capacity measured via ADP/O-GM and ADP/O-succinate to 55% and 52% in group SRM1day_IR compared to normal. Administration of GSK3β inhibitor improved the mitochondrial respiration capacity by 47% (GM medium) and 26% (succinate medium) in SRM1day + SB216763_IR (vs. group SRM1day_IR). Similarly, the respiratory control ratio that determines the coupling efficiency showed a significant decline of 60% (GM medium) and 38% (succinate medium) in SRM1day_IR (vs. normal), which was increased to 39% and 17% in the presence of GSK3β inhibitor in SRM1day + SB216763_IR (vs. SRM1day_IR). The ATP levels showed a significant decline of 48% in SRM1day_IR; however, IR-induced PM_2.5_-exposed hearts showed a 38% elevation in the ATP levels after the administration of SB216763.

### 3.9. Protein Expression Level of p-GSK3β and GSK3β in Cardiac Tissue and Mitochondria from Rats Exposed to PM_2.5_ for 1 Day in the Presence and Absence of GSK3β Inhibitor

**Figure 10:**
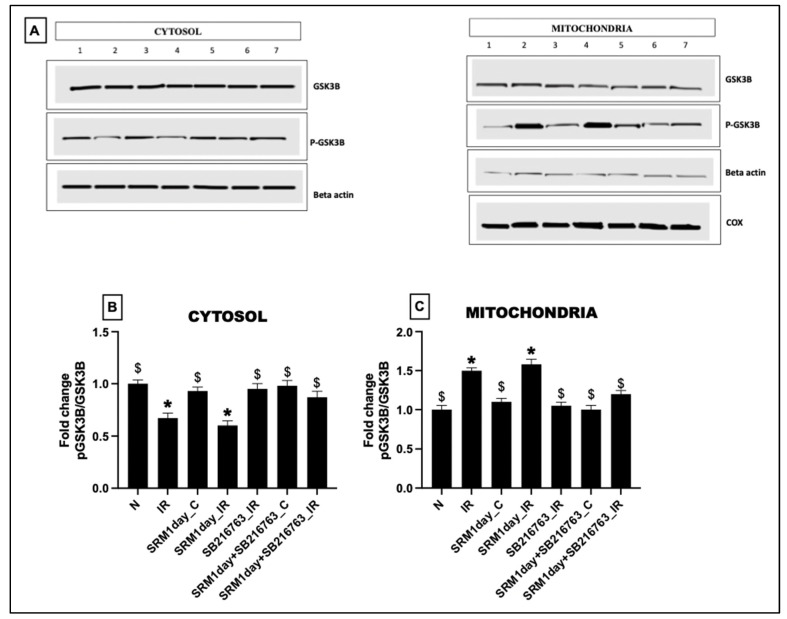
(**A**) Protein expression of GSK3β and p-GSK3β. Quantification of proteins in (**B**) cytosol and (**C**) mitochondria (* *p* < 0.05 vs. N, ^$^ *p* < 0.05 vs. IR). Groups 1: Animals underwent normal perfusion (normal), 2: animals underwent the standard IR procedure of 30 min of ischemia and 60 min of reperfusion (IR control), 3: animals were exposed to PM_2.5_ for 21 days after which the hearts were isolated and subjected to normal perfusion, 4: animals were exposed to PM_2.5_ for 1 day after which the hearts were isolated and subjected to IR procedure, 5: animals were administered SB216763 intraperitoneally after which the hearts were isolated and subjected to IR procedure, 6: animals were exposed to PM_2.5_ for 1 day and administered SB216763 intraperitoneally after which the hearts were isolated and subjected to normal perfusion, and (7) animals were exposed to PM_2.5_ for 1 day and administered SB216763 intraperitoneally after which the hearts were isolated and subjected to IR procedure (6 animals in each group).

As observed in Figure 10, relatively similar band intensity for total GSK3β was observed across different experimental groups in both cytosol and mitochondrial fractions separately. However, the p-GSK3β was relatively low in the cytosolic fraction of IR tissue from groups 2 (IR) and 4 (SRM1day_IR). A corresponding increase in p-GSK3β was noted in groups 2 (IR) and 4 (SRM1day_IR) in the mitochondrial fraction, signifying the translocation of p-GSK3β from the cytosol to mitochondria and indicating the stress experienced by the mitochondria. However, in the presence of a GSK3β inhibitor, translocation of p-GSK3β into mitochondria was negligible in SB216763_IR and SRM1day + SB216763_C, suggesting an active mitochondrial pool that enables the regulation of cellular metabolism in response to ischemia–reperfusion.

## 4. Discussion

GSK3β is reported to play an essential role in the pathophysiology of IR. Early studies have reported the cause–effect relationship of GSK3β in IR pathology, where the underlying mechanism was reported to be associated with the preservation of mitochondrial activities and prevention of cell death pathways [11,21]. Essentially, the GSK3β pool of mitochondria is found to regulate various mitochondrial events that include bioenergetics function, biogenesis, permeability, motility, and apoptosis. Mitochondrial dysfunction is one of the main hallmarks of IR pathology [30], and considering the role played by GSK3β in both IR injury and mitochondrial function, we explored whether GSK3β can be a promising therapeutic target in the management of IR in an experimental set up of rats exposed to PM_2.5_ for 21 days. The major findings derived from the present study include (i) SB216763 was effective in attenuating IR injury and improved physiological recovery of isolated rat hearts only when the rats were exposed to PM_2.5_ for 3 h by a single exposure. However, this protective effect was negated in isolated rat hearts from animals exposed to PM_2.5_ for 3 h daily for 21 days. (ii) Stress-based translocation of GSK3β protein into mitochondria was found in PM_2.5_-exposed rat hearts and its level in mitochondria increased substantially with IR induction. GSK3 β inhibitor could able to prevent its translocation only in IR control (not exposed to PM_2.5_) rat hearts. In non-mitochondrial (cytosolic fraction) samples, GSK3β protein level was low in PM_2.5_-exposed rat hearts, which was increased with SB216763 administration in control hearts, but not in PM_2.5_-exposed rat hearts. Incidentally, IR induction further deteriorated the GSK3β protein expression, thereby being unable to activate the downstream cardio-protective mediator, especially mitochondrial function. (iii) Accumulation of PM_2.5_ components in the mitochondria (measured via ICPMS) not only declined the respiratory function and ATP production but also enhanced the oxidative stress and downregulated the expression of mitochondria-specific antioxidant genes like glutaredoxin and peroxiredoxin in the hearts of rats exposed to PM_2.5_ for 21 days. The addition of a GSK3β inhibitor did not reverse these changes effectively. However, with IR induction in PM_2.5_-exposed hearts for 21 days, these changes further worsened. (iv) The GSK3β inhibitor was unable to improve the expression of mitochondrial quality control genes, significantly contributing to the negative effects observed in rat hearts exposed to PM_2.5_ for 21 days and challenged with IR. (v) Contrary to the above findings, single-day-exposed rat hearts effectively improved oxidative stress and mitochondrial genes with GSK3β inhibitor treatment. (vi) Twenty-one days of PM_2.5_ exposure decreased the mitophagy gene expression (maybe the effect of metal deposition), which in turn adversely affects the overall mitochondrial functional activity. These findings indicate that mitochondrial functional recovery events via activating the adaptive response were intact with a single 3 h exposure to PM_2.5_. (vii) Heavy metal binding proteins like metallothionine gene expression was significantly elevated in myocardial tissue from rats exposed to PM_2.5_, indicating the accumulation of metals in mitochondria which was confirmed by ICPMS analysis. Interestingly, its expression significantly increased with IR challenge to PM_2.5_-exposed rat hearts due to the stress induced by metals.

GSK3β has shown therapeutic promise in numerous preclinical trials, most of which used normal animals to treat different diseases [31,32]. Furthermore, a few recent studies demonstrated the protective effect of GSK3β inhibitors in attenuating PM_2.5_-induced inflammatory response in bronchial epithelial cells by regulating JNK/NF-KB pathways [33]. Through activating the master regulatory protein, peroxisome proliferators activated receptor gamma co-activator 1 alpha (PGC-1α), which orchestrates mitochondrial biogenesis and controls bioenergetics. GSK-3β signaling plays a crucial role in regulating the functions of the mitochondria [21]. Cardiac physiological function is closely related to mitochondrial bioenergetics function, mitochondrial dynamics, and copy number [34]. The majority of cardiac anomalies are linked to mitochondrial dysfunction that deprives energy and promotes the accumulation of ROS and dysregulation of ionic and metabolic balance [35]. Thus, targeting to maintain mitochondrial function is considered to be one of the promising strategies in the management of cardiac vascular diseases [36], where many investigators identified GSK 3β as a potential and promising target in the treatment. Through the present study data, we demonstrated that GSK 3β may not be an efficient target in attenuating myocardial ischemia–reperfusion injury in PM_2.5_-mediated modified hearts. Recent studies have affirmed that PM_2.5_ from the air samples can inflict/promote cardiac disease through structural modifications in the heart and vasculature [37]. Thus, exposure to pollutant air can substantially contribute to the development of ischemic heart diseases, where the patients are often treated with revascularization procedures [38]. This surgical procedure may induce unavoidable injury termed reperfusion injury, leading to many post-operative complications and deteriorated rehabilitation efficiency. The present study outcome suggests that PM_2.5_ exposure depleted the cardiac tolerance to withstand additional surgical stress, which makes the cardiac patients highly sensitive to developing co-morbidity or even inflicting mortality with surgery. Another insightful meaning of the findings suggests that signaling therapeutic targets for the treatment of IR injury may not be effective if the majority of the cardiac mitochondria are not healthy. Through this study, we showed that PM_2.5_ exposure instigates mitochondrial damage via considerable uptake of PM_2.5_ by the organelle, which leads to depleted mitochondrial quality control, eventually causing cardiac injury and deteriorated contractile function of the heart. In the presence of a low level of healthy mitochondria in the cardiac tissue, the GSK 3β inhibitor is unable to provide the desirable function of protecting the heart from IR injury.

## Figures and Tables

**Table 1:** List of primers used in the course of the study.

S No:	Gene	Forward Primer	Reverse Primer
1	PGC-1α	5′-GAGGGACGAATACCGCAGAG-3′	5′-CTCTCAGTTCTGTCCGCGTT-3′
2	Dnm1	5′-TTGCCCTCTTCAACACTGAGC-3′	5′-ATGAAGCTGTCAGAGCCGTT-3′
3	Parkin	5′-AGTTTGTCCACGACGCTCAA-3′	5′-CAGAAAACGAACCCACAGCC-3′
4	MFN1	5′-TGACTTGGACTACTCGTGCG-3′	5′-GGCACAGTCGAGCAAAAGTG-3′
5	MFN2	5′-CTCTGTGCTGGTTGACGAGT-3′	5′-TCGAGGGACCAGCATGTCTA-3′
6	DRP1	5′-TGGAAAGAGCTCAGTGCTGG-3′	5′-TCAACTCCATTTTCTTCTCCTGT-3′
7	MFF	5′-GAAAACACCTCCACGTGTGC-3′	5′-CTGCTCGGATCTCTTCGCTT-3′
8	FIS	5′-CCAGAGATGAAGCTGCAAGGA-3′	5′-TTCCTTGAGCCGGTAGTTGC-3′
9	PINK1	5′-TGTATGAAGCCACCATGCCC-3′	5′-TCTGCTCCCTTTGAGACGAC-3′
10	TFAM	5′-GTTGCTGTCGCTTGTGAGTG-3′	5′-GTCTTTGAGTCCCCCATCCC-3′
11	β-actin	5′-GTGTGGTCAGCCCTGTAGTT-3′	5′-CCTAGAAGCATTTGCGGTGC-3′
12	POLG1	5′-CTTTGGGCTCCAGCTTGACT-3′	5′-TGGAGAAAATGCTTGGCACG-3′
17	Glutaredoxin1	5′-AGCATGGCTCAGGACTTTGT-3′	5′-TTGAATCGCATTGGTGTTGT-3′
18	Peroxiredoxin 6	5′-CCTGGAGCAAGGACATCAAT-3′	5′-GTTTCTTGTCAGGGCCAAAA-3′
19	Peroxiredoxin 3	5′-CCAGAGTCCCCTACGATCAA-3′	5′-TGGCCACCTTTAACCTGAAC-3′
20	D2HGDH	5′-ATCATGCCACTGGACTTGGG-3′	5′-AGCAAGTACCACTTCCAGGC-3′
21	ECHS1	5′-GCCTCGGGTGCTAACTTTCA-3′	5′-GGTCTCCAGTGCTTGGTTGA-3′
22	ETHE1	5′-GCTTTCACTGGAGATGCCCT-3′	5′-GAAACTGTGAGCCCGTGGTA-3′
23	HIBCH	5′-TGGAATCACAATGGGTGGGG-3′	5′-ATCCTCAGCTGAAGGGGACT-3′
24	L2HGDH	5′-GTCCAAGGCTTGAGGCTGAT-3′	5′-GGGCAAATGACAACGCTACC-3′
25	SLC25A1	5′-GGGAAAGCCAAACTGACGC-3′	5′-TCTTCACGTATTCGGTCGGG-3′
26	TXN2	5′-TTCCCTCACCTCTACGAGCC-3′	5′-GGTGGTGTGAAATGTCCGGG-3′
27	TXNIP	5′-AACCCACTCGGCTCAATCAT-3′	5′-CCGCTGCCATACACCTTCTC-3′
28	Metallothionine 1	5′-CTCTCCTCACAGGGTAGGGT-3′	5′-GGCGGGAATAGGCACCTTTA-3′
29	Nrf2	5′-AGGTTGCCCACATTCCCAAA-3′	5′-ATATCCAGGGCAAGCGACTG-3′

## Data Availability

Data will be made available upon request.

## Supplementary Materials

The following supporting information can be downloaded at https://www.mdpi.com/article/10.3390/life13112234/s1.

## Abbreviations

PM: particulate matter; DPM: diesel particulate matter; GSK3β: glycogen synthase kinase-3 beta; IR: ischemia–reperfusion injury; ROS: reactive oxygen species; ETC: electron transport chain; KH: Krebs–Henseleit buffer; RCR: respiratory control ratio; GSH: reduced glutathione; GSSG: oxidized glutathione.
