# Supplementary material for: PM2.5 Exposure-Linked Mitochondrial Dysfunction Negates SB216763-Mediated Cardio-Protection against Myocardial Ischemia–Reperfusion Injury
Source: Life (Basel). 2023 Nov 20;13(11):2234. doi: 10.3390/life13112234 (PMC10672572; doi:10.3390/life13112234)
Supplement: Supplementary file 1 [file life-13-02234-s001.zip › life-2672295-supplementary.pdf]

Figure:3

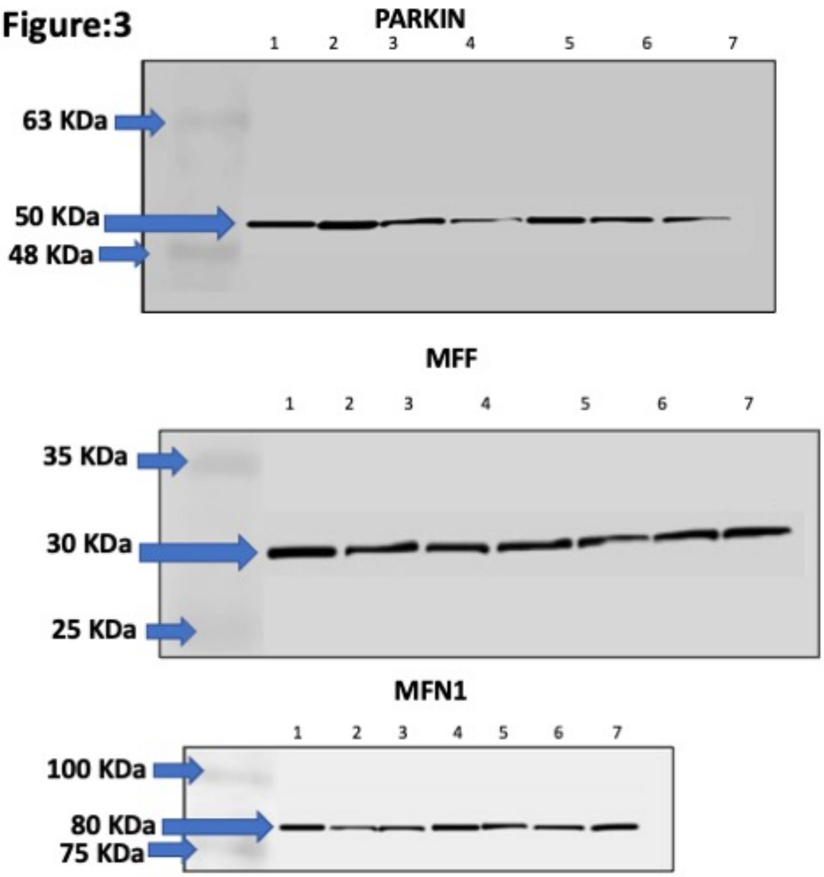

Figure:3

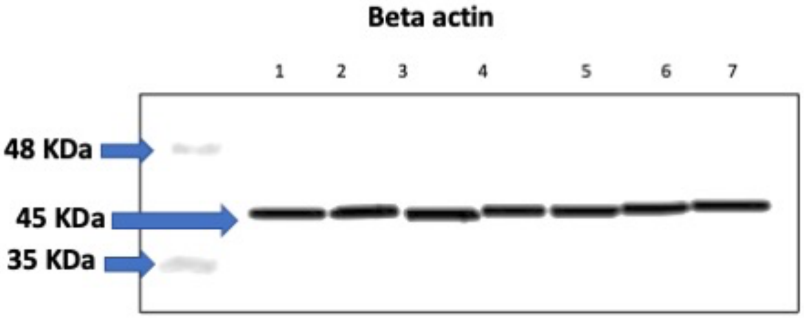

Figure S1: Showing the original western blot images for Figure 3 with molecular weight markers. The quantification of bands is displayed in the manuscript file.

Figure:7

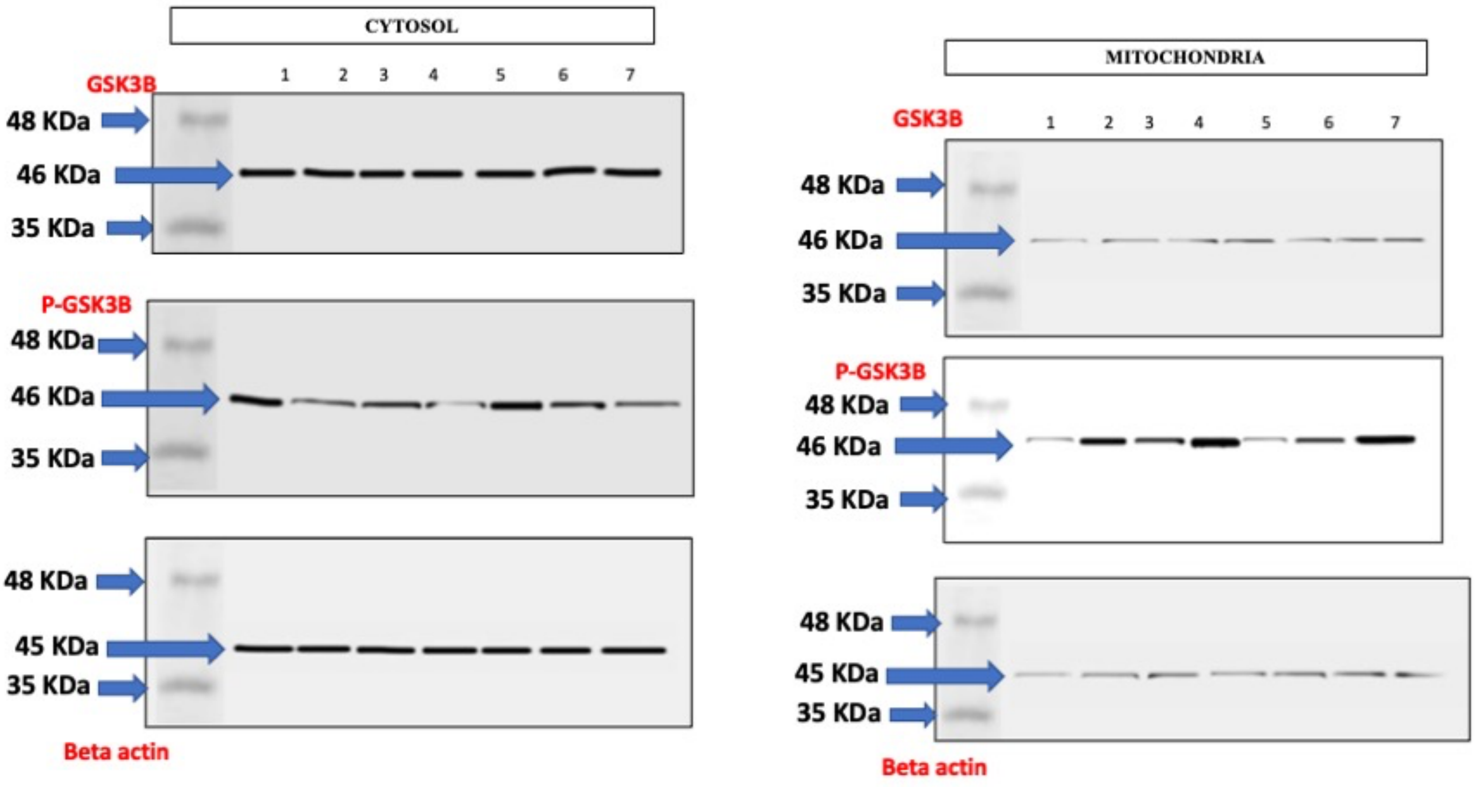

Figure:7

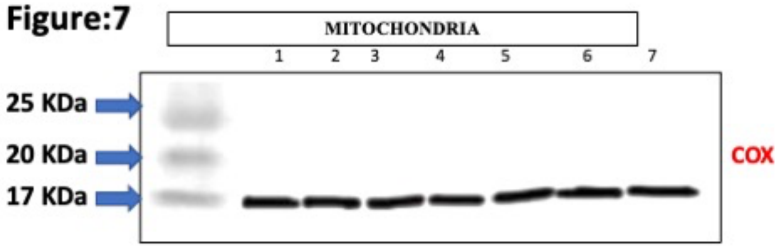

Figure S2: Showing the original western blot images for Figure 7 with molecular weight markers. The quantification of bands is displayed in the manuscript file.

Figure:9

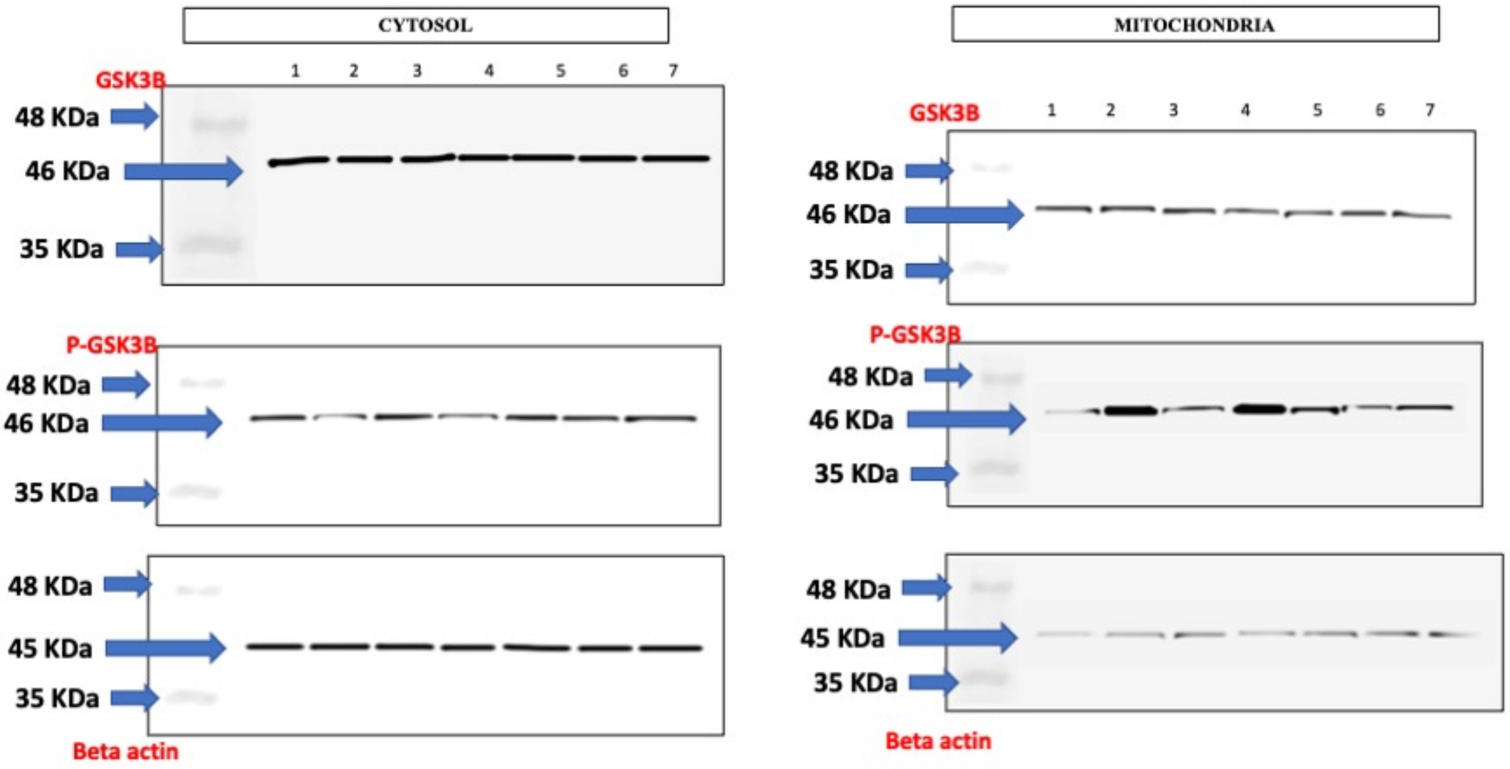

Figure:9

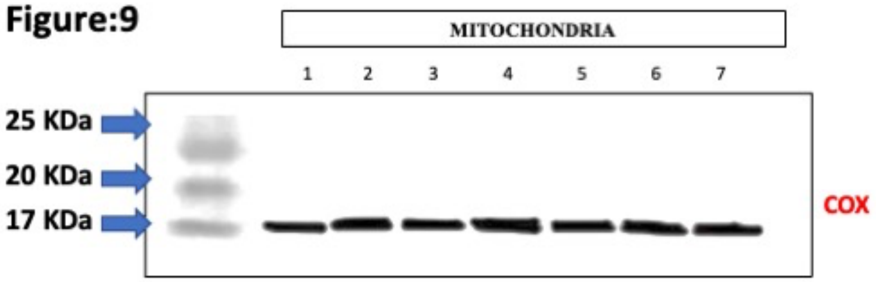

Figure S3: Showing the original western blot images for Figure 9 with molecular weight markers. The quantification of bands is displayed in the manuscript file.
